# Pelvic joint fusion in patients with severe pelvic girdle pain – a prospective single-subject research design study

**DOI:** 10.1186/1471-2474-15-85

**Published:** 2014-03-15

**Authors:** Thomas J Kibsgård, Olav Røise, Britt Stuge

**Affiliations:** 1 Department of Orthopaedics, Division of Surgery and Clinical Neuroscience, Oslo University Hospital, Sognsvannsveien 20, 0372 Oslo, Norway; 2 Institute of Clinical Medicine, Faculty of Medicine, University of Oslo, Oslo, Norway

**Keywords:** Sacroiliac joint, Fusion, Pain, Arthrodesis, Surgery, Pelvic girdle pain

## Abstract

**Background:**

The fusion of the pelvic joints in patients with severe pelvic girdle pain (PGP) is a controversial and insufficiently studied procedure. The aims of this study were to evaluate physical function and pain after sacroiliac joint (SIJ) fusion.

**Methods:**

A single-subject research design study with repeated measurements was conducted; pre-operatively and at 3, 6 and 12 months post-operatively. The outcome measures considered were the Oswestry disability index (ODI), visual analogue scale (VAS), and SF-36. Eight patients with severe PGP received open-accessed unilateral anterior SIJ fusion and fusion of the pubic symphysis.

**Results:**

Seven patients reported positive results from the surgery. At 1 year post-operation, significant (p < 0.001) reductions in ODI (54 to 37) and VAS (82 to 57) were reported. The physical functioning, bodily pain, and social functioning scores in the SF-36 were also improved.

**Conclusion:**

Positive and significant changes in disability and pain at 1 year after SIJ fusion were observed. Despite these positive results, open accessed anterior fusion of the SIJ was associated with adverse events and complications such as infection and nerve damage.

## Background

The sacroiliac joint (SIJ) may be the source of pain for 13-30% of patients with low back pain [1], and possibly an even higher proportion of patients suffering from “failed back surgery” [2,3]. This pain may be caused by specific pathology of the joint [4], but the specific role of the SIJ in unspecific pelvic girdle pain (PGP) disorder remains unknown. PGP is a common complaint in pregnancy that might cause disability, and in some women the complaint continues after delivery [1,5]. The origin and diagnosis of PGP are also unclear, as radiological findings are absent and the diagnostic criteria lack sufficient evidence. However it has become increasingly clear that patients with PGP have a different clinical presentation than patients suffering from low back pain [6]. Based on the theory of pathological joint mobility, SIJ fusion associated with symphysis pubis fusion is a therapeutic option when conservative treatment is unsuccessful [7].

SIJ fusion was first described by Smith-Petersen in 1921 [8]. Pelvic joint fusion has since been reported in a number of studies, but there is limited evidence in support of its efficacy [9-13]. The results of pelvic joint fusions have mostly been reported in small case series, with the exception of one study that included 58 patients, however without a control group [12]. The reported short-term results have been mainly positive [9,10,12,13], but poor results have also been reported [11]. One recent study showed that among patients with successful short-term outcomes, the effect was sustained 23 years post-operatively [14].

A randomized controlled trial is the gold standard to examine the effect of an intervention. As SIJ fusion is performed on few patients, a single-center randomized controlled design is difficult to establish due to the low number of participants. However single subject research design (SSRD) have been recommended as a useful method to examine clinical accountability [15]. If properly applied, a SSRD can provide a systematic approach to documenting clinical change, and also provide objective evidence regarding the efficacy of a treatment modality [15]. SSRD refers to a study of a single patient or a small number of patients, observed over time, in which the treatment and outcome variables are controlled. The design comprises of multiple measurements before (baseline), and at different phases after, the intervention [15,16]. SSRD focuses on individual responses and repeated measurements that improve the validity of the study. When the SSRD is replicated across patients, the internal and external validity is strengthened and allows inferences to be made about effectiveness.

The primary aim of this prospective study was to examine changes in pain and physical function at 3, 6, and 12 months after SIJ fusion. The secondary aims were to evaluate post-operative health-related quality of life and patient satisfaction with treatment.

## Methods

**Figure 1:**
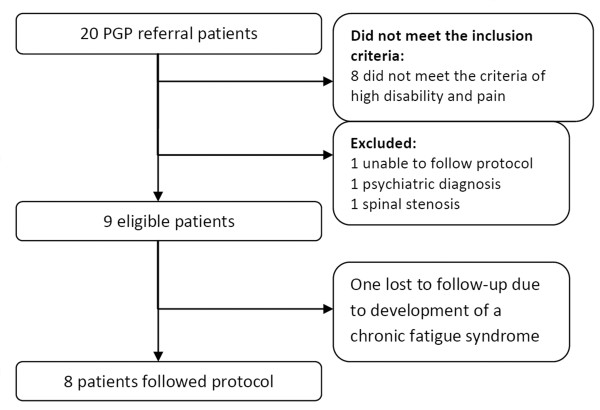
Flow-chart of the study patients.

During the study period, from 2007 and 2010, a total of 20 patients with PGP were referred to our pelvic centre, but only 9 patients met the study’s inclusion criteria (Figure 1). A SSRD was used to evaluate the outcomes for pain, disability and health-related quality of life [15,16]. Five data collection sessions were conducted in each of the following 4 phases: prior to surgery (baseline) and at 3, 6, and 12 months after surgery. Inclusion and exclusion criteria are presented in Table 1. Based on previous findings that female patients with PGP have variations in pain intensity during their menstrual cycle [17], the patients filled out a questionnaire every Thursday for 5 weeks in each phase to ensure that evaluations were made throughout the menstrual cycle. The questionnaires were returned weekly by mail. All patients underwent 3 clinical examinations and CT guided SIJ injections before the decision for SIJ fusion was taken. The CT guided injections were performed by two experienced radiologists, and the patients filled out a VAS scale before and 2 hours after the injection.The patients received surgery to fuse the most painful SIJ, and the pubic symphysis was fused in all cases. The fusions were evaluated with a CT scan at 1-year follow-up.

**Table 1:** Inclusion and exclusion criteria

**Inclusion criteria**	**Exclusion criteria**
1. Pain located to one or more pelvic joints	1. Known psychiatric diagnosis,
2. Minimum two positive out of five clinical tests:	2. Other spine pathology
*Posterior Pelvic Pain Provocation test (P4)	3. CT verified ankylosis at baseline
*Active Straight Leg Raise (ASLR)	4. Body mass index over 30
*Palpation of the long dorsal SI-ligament	
*Modified Trendelenburg test	
*Palpation of the symphysis	
3. High pain and disability score	
*Oswestry Disability Index >40 and/or	
*Visual Analogue Scale >50	
4. The patients should have performed adequate physiotherapy over time without positive effect	

*One could not perform the test.

All patients signed a written informed consent for participation. The project was approved by the Regional committees for medical and health research ethics, Region South East, Norway (number: 1.2006.1574) and registered in the Clinical Trials Database (reference number: NCT00900601).

### Outcome measurements

**Figure 2:**
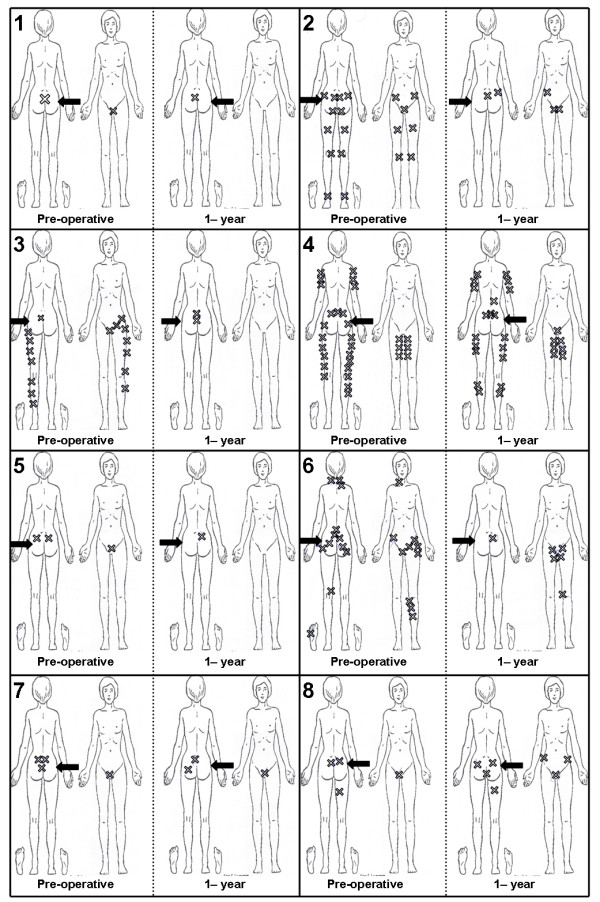
**Pre-operative and post-operative pain diagrams for each of the eight patients.** The arrow represents the operated side.

Function was measured according to the Oswestry disability index (ODI) [18], each patient’s most severe morning and evening pain intensity was assessed using a 100 mm visual analogue scale (VAS) (0 = no pain, 100 = worst possible pain), and health-related quality of life was assessed using the SF-36 [19]. The ODI is a 10-item questionnaire that assesses function (0–100, with lower scores indicating less disability), for which a 10-point difference represents a significant clinical change [18,20]. In addition to the VAS, diagrams were used to record pain localization (Figure 2). The SF-36 questionnaire contains 36 items representing 8 subscales, including physical functioning, role limitations due to pain, bodily pain, general health, vitality, social functioning, role limitations due to emotional challenges, and mental health. The SF-36 scores are transformed to a 0–100 scale for each subscale. The higher the score, the better the health status. Additionally, the patients answered the following two questions: “Have you experienced any effect of the surgery? If so, would you grade this as excellent, good, some, minor or no effect?” and “How do you tolerate physical activity now, as compared to before surgery?”

### Surgical procedure

**Figure 3:**
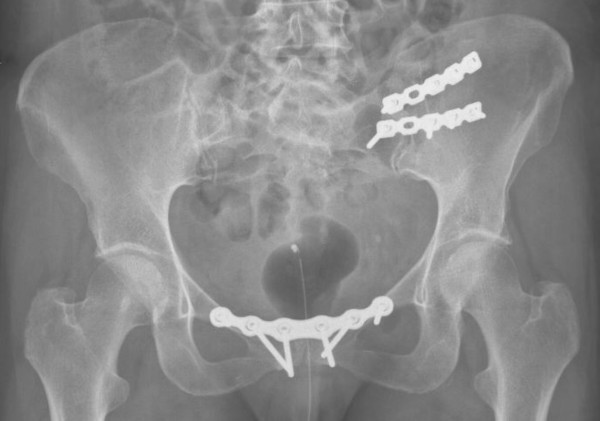
Post-operative X-ray of SIJ fusion with concomitant fusion of the pubic symphysis.

The patients received unilateral SIJ fusion, of the most painful SIJ, and symphysiodesis. An anterior approach with a skin incision over the iliac crest was used to reach the SIJ. The joint was partially resected, and grafted with cancellous bone from the ipsilateral iliac crest. Two AO reconstruction plates or AO-DC plates (Synthes®, Synthes GmbH, Switzerland) were used (Figure 3) to achieve stabilisation. The pubic symphysis was accessed through a bikini line incision. A 2 × 2 cm bone block was removed and replaced with a bone graft from the iliac crest, and a Matta plate (Stryker®, Michigan, United States) was applied (Figure 3). Post-operatively, the patients received epidural anaesthesia pain relief and 1–2 days of wound drainage. The patients were advised to avoid full weight bearing activities, on the operated side, for 8 weeks after the surgery.

### Data analysis

The graphed data were analysed according to the guidelines for SSRD [16]. The levels (mean measurements over the 5-week period) and variability of the measurements are presented graphically. To analyse the changes in ODI, VAS, and SF-36, a mixed model for repeated measurements was used. The statistical analyses were performed using STATA 12.0 (Statacorp, Texas, USA). ODI, VAS, and individual items of the SF-36 were used as dependent variables, and time was used as an independent variable. This provided a regression line = constant value (baseline) + regression coefficient × time, where time was defined as either 0 = baseline, 1 = 3 months, 2 = 6 months or 3 = 1 year. The correlation structure was specified as independent, and the regression slopes were allowed to vary at random. The correlation matrix was also tested as unstructured, but this did not alter the regression slope. We considered differences significant if the p value was less than 0.05.

## Results

Nine consecutive patients received unilateral anterior SIJ fusion and fusion of the pubic symphysis. One patient developed chronic fatigue syndrome during the follow-up and dropped out of the study after 6 months. The remaining eight patients followed the study protocol; the baseline characteristics of these patients are presented in Table 2. Seven of the patients reported bilateral SIJ symptoms, mostly marked on one side, and six of them also had pain in the pubic symphysis. One patient reported unilateral SIJ pain and pain in the pubic symphysis. All patients had CT guided injection before surgery. Five of these patients experienced more than 70% pain relief from the injection (patient 1, 4, 6, 7 and 8) and 3 patients (2, 3 and 5) experienced no effect from the injections.

**Table 2:** Patient characteristics pre-operatively

	**Mean**	**Range (min-max)**
Age (years)	40	(33–47)
Sex: female/male	8/0	
BMI (kg)	25	(20–30)
Children	2.8	(0–5)
Duration of symptoms (years)	11	(2–25)
Disability pension		
*• 100%*	3	
*• Graded*	1	
Sick leave		
*• 100%*	3	
*• Graded*	1	
Unilateral/Bilateral SIJ symptoms	1/7	
Pain in the pubic symphysis	7	
Etiology		
*• Post pregnancy*	6	
*• Trauma*	2	
Positive clinical test		
*1. Posterior pelvic provocation test*	6	
*2. Active straight leg raise*	8	
*3. Modified Trendelenburg*	4*	
*4. Pain while palpation over the long dorsal ligament*	8	
*5. Palpation of the symphysis*	7	

*One could not perform the test.

**Figure 4:**
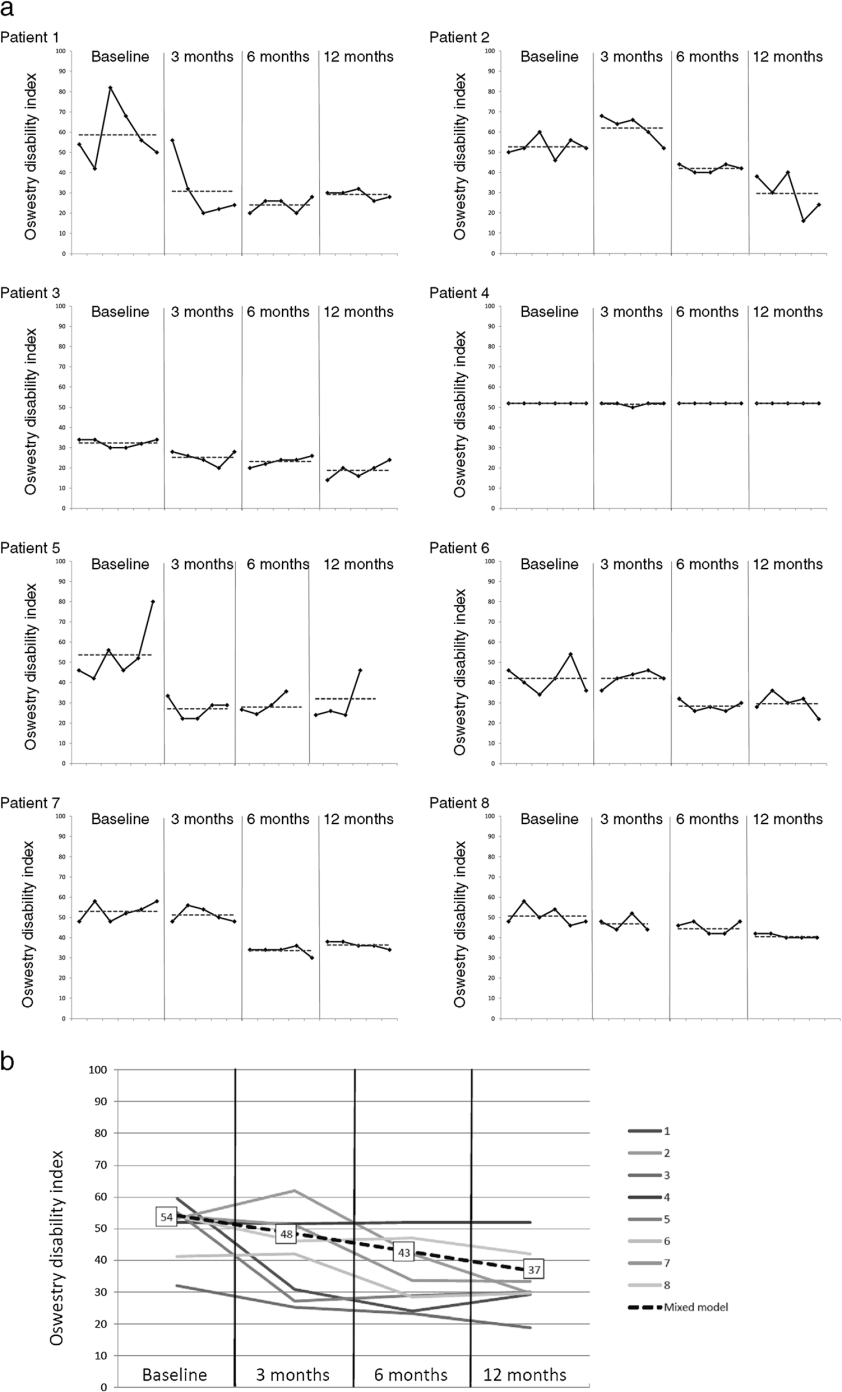
**Results of changes in oswestry disability index. a)** Functional status. The Oswestry disability index (ODI; 0–100, with lower scores indicating less disability) values are presented as a single-subject design study graph of each individual patient. Repeated measurements in each of the four different phases — baseline (pre-operative) and 3 months, 6 months and 12 months post-operatively — are presented (black line). The mean value of each phase is indicated by a dotted line. **b)** The mean ODI of each individual patient is presented, together with the regression line from the mixed model (ODI = 54.2 - 5.7 × time).

The ODI scores of each patient are presented in Figure 4. All but one patient exhibited a decrease of more than 10 points on the ODI from the pre-operative period to the 1-year follow-up. One patient experienced no effect. There was a strong association between ODI and time, with a 17-point decrease (p < 0.001) at 1 year after surgery (Table 3, Figure 4). The graphs show significant variations in the measurements at each time point. At baseline, a difference of more than 40 points between the maximum and minimum values was observed in two patients, and only two patients had less than a 10-point difference.

**Table 3:** Mixed model for repeated measurements

	**Pre-operative score constant value (95% CI)**		**Regression coefficient (95% CI)**			**Score at 1-year follow-up**
ODI	54.2	(48.4-59.9)	-5.7	(-7.6- -3.8)	*p < 0.001*	37.1
VAS in the morning	59.5	(45.6-73.5)	-4.8	(-9.0- -0.7)	*p = 0.019*	45.1
VAS in the evening	81.7	(76.3-87.2)	-8.4	(-12.3- -4.5)	*p < 0.001*	56.5
SF-36						
Physical functioning	21.5	(8.8-34.3)	7.0	(3.2-10.8)	*p < 0.001*	42.5
Role physical	2.8	(-6.7-12.3)	2.6	(-1.1-6.3)	*p = 0.169*	10.6
Bodily pain	13.1	(4.7-21.6)	6.8	(4.2-9.4)	*p < 0.001*	33.5
General health	48.4	(34.9-61.9)	2.0	(0.5-3.5)	*p = 0.009*	54.4
Vitality	42.8	(33.3-52.4)	1.5	(-0.7-3.6)	*p = 0.174*	47.3
Sosial functioning	41.8	(22.4-61.2)	5.2	(1.3-9.0)	*p = 0.008*	57.4
Role emotional	55.1	(24.8-86.2)	2.6	(-1.7-7.0)	*p = 0.240*	62.9
Mental health	75.1	(64.1-86.1)	-1.0	(-2.7-0.7)	*p = 0.224*	72.1

ODI, VAS and individual items of SF-36 as dependent variables and time as independent variable. Regression line: constant + regression coefficient × time (time is defined as; 0 = baseline, 1 = 3 months, 2 = 6 months, 3 = 12 months). Score at 1-year follow-up = constant value at baseline + 3 times regression coefficient.

**Figure 5:**
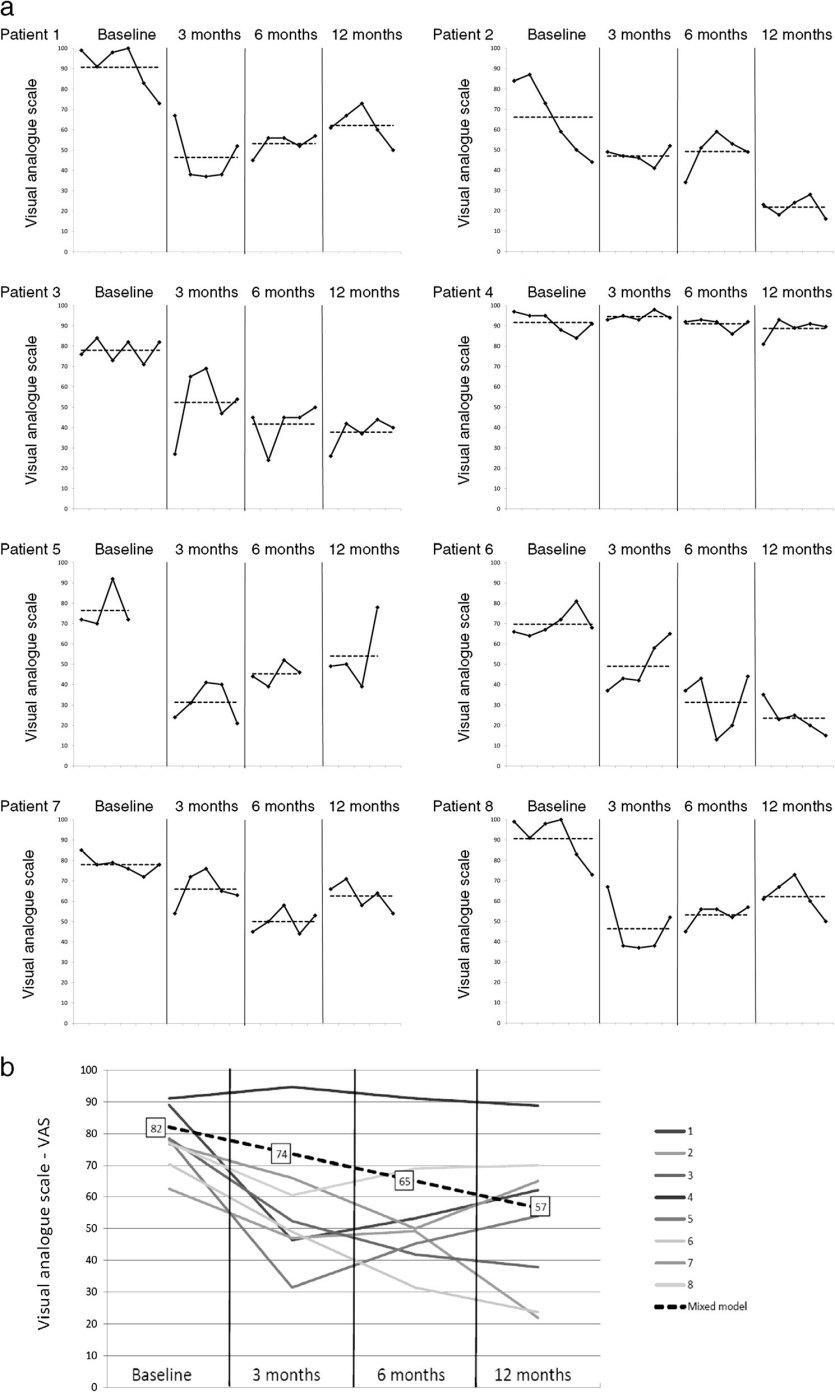
**Results of changes in pain using the visual analogue scale. a)** Pain intensity. Visual analogue scale (VAS; 0-no pain, 100-worst pain) values of evening pain, presented as a single-subject design study graph for each individual patient. Repeated measurements in each of the four different phases — baseline (pre-operative) and 3 months, 6 months and 12 months post-operatively — are presented (black line). The mean value of each phase is indicated by a dotted line. **b)** The mean VAS of each patient is presented, together with the regression line from the mixed model (VAS = 81.7 - 8.4 × time).

The VAS scores of each patient are presented in Figure 5. The patients showed a reduction in pain, with a decrease from 82 points at baseline to 57 points after 1 year (p < 0.001, regression coefficient of -8.4) (Figure 5, Table 2). All patients reported a decrease in pain. The difference between the pre-operative status and the 1-year follow-up scores was greater than 40 points in three patients, between 22 and 29 in three patients, and 15 points in one patient. One patient had only a minor change (3 points); although she had a positive SIJ injection she showed a more generalised pain pattern than the other patients (Figure 2). Pre-operatively, a difference of 43 points between the maximum and minimum scores was observed in one patient, and no patient had variations of less than 10 points.

At baseline, seven out of eight patients had bilateral SIJ symptoms. At the 1-year follow-up, only two patients experienced pain in the fused joint; however, six of the seven patients reported discomfort in the contralateral side. Seven patients had pain in the pubic symphysis before surgery, and five still had pain in this area at the 1-year follow-up (Figure 2).

The patients showed low health-related quality of life scores at baseline as compared to the general Norwegian population [19]. These patients also scored lower on the physical items of the SF-36 than on the items covering mental health. One year after surgery, there was a 20-point improvement in physical function and bodily pain (p < 0.001), a 15-point improvement in social functioning (p = 0.008) and a 6-point improvement in general health (p = 0.009) (Table 3).

All patients reported that surgery had a positive effect; one patient reported a minor effect, two reported some effect, and five reported a good effect from the surgery. None of the patients reported an excellent result. Concerning tolerance of physical activity, seven patients reported some improvement, and one patient reported major improvement.

The fusion was evaluated with CT at the 1-year follow-up, and all patients had either solid fusion or significant bone bridging in the SIJ. However it was difficult to evaluate the fusion in the pubic symphysis because of the plate artefacts, but no patient had plate or screw loosening or other signs of non-union.

There were 3 major complications: one infection, one complex regional pain syndrome with drop-foot and one loss of bladder sensation. In addition there were 3 patients with transient sensitivity loss to the lateral femoral cutaneous nerve, a possible complication of bone harvesting from the iliac crest. All patients reported high post-operative pain and required epidural treatment for 5–7 days. They were hospitalised for 7–10 days and were discharged with opioids.

## Discussion

The primary aims of this study were to evaluate changes in disability and pain intensity after SIJ fusion in patients with severe PGP. Pre-operatively, these patients showed severe disability and high pain levels. One-year post-operatively, clinically significant reductions in both disability and pain were observed. The SF-36 scores for physical function, bodily pain and social functioning also improved significantly. Seven out of eight patients reported a positive effect from the procedure.

Pelvic fusion in PGP is a rare procedure and is only performed in severe cases where conservative treatment modalities have been unsuccessful. A randomized controlled trial of this procedure is not possible, as the alternative treatment modality (conservative treatment) has already been tried. For this reason, an alternative study design was sought. The SSRD with multiple measurements is designed to study small samples of patients. A sample size of three patients is considered sufficient for external validity [15,16] and a study with 8 patients is scientifically generalizable. Patients with PGP have reported cyclic variations in symptoms, and as many as 72% report relapses during menstruation [17]. To capture these potential variations we repeatedly collected patients’ data for a 5-week period and discovered a large variation in the values during each phase. For some patients at baseline, a 40-point difference in ODI and a 43-point difference in VAS were observed during the 5-week period. One strength of the SSRD is its ability to uncover these individual variations, which is important for studying patients with PGP. Some of these variations may be corrected for in large group studies, but conclusions from small case series, with single measurements, should be interpreted with caution. A limitation of our study is the short follow-up period of 1 year. Although a 1-year follow-up period for clinical trials is commonly regarded as being too short, a recent study showed that the 1-year outcome after SIJ fusion was sustained 23 years later [14]. Despite the limitations of the SSRD, we believe that our study contributes valuable information on the effects of pelvic joint fusion.

Outcomes for SIJ fusion have been reported in several case series [9-14]. A positive effect of the surgery was observed in 50% to 90% of patients and this is in accordance with the positive effects seen in our 1-year outcomes. In a case series of nine patients, Al-Khayer et al. [9] observed decreases from 59 to 45 for mean ODI (p < 0.005) and from 8.1 to 4.6 for mean VAS (0–10) (p < 0.002). The same positive outcomes were reported by van Zwienen et al. [12], who found that 58 patients exhibited an increase in physical outcome from 37 to 61 (p < 0.001) as measured using the Majeed score (0-poor, 100-good) [21]. Although a mean improvement in physical function was observed in this study, 27% of patients reported a poor result with no effect from the surgery. Most of these patients had complications or non-union events, but some experienced no effect without any proper explanation. In our study, one patient did not experience any effect from the surgery. This patient had a more generalised pain pattern than the others (Figure 1) and it is possible that the SIJ was not the major source of pain in this case, although she had a positive response to the SIJ injection. In contrast the patients who reported a sharp and localised pain in the SIJ area did benefit from surgery. Hence a major challenge for clinicians is to identify patients who could possibly benefit from surgery. In our study, seven out of eight patients had a positive effect, indicating that our patient selection criteria were reasonably successful. However further studies must be conducted to identify the optimal criteria for the identification of patients to be offered surgical treatment.

Surgery is generally associated with a risk of complications. Because of the location of the SIJ an open approach to this structure is quite an aggressive surgical procedure. One of our patients developed a complex regional pain syndrome despite displays of normal neurological function in the first two post-operative days. This phenomenon has been found to occur after anterior SIJ fusion and is most likely due to nerve root compression [22]. When performing SIJ fusion the most serious and common complications are non-union, infection and nerve damage [10,12]. Van Zwienen et al. operated on 58 patients with bilateral SI screws and plating of the pubic symphysis and reported a 46.6% complication rate [12]. Patients experiencing complications report poorer outcomes than those without complications [10-13]. In our study, three patients experienced a major complication or adverse event but still reported satisfaction with the surgery because their SIJ pain had been relieved.

We fused the pubic symphysis for every patient based on experience that this procedure increases pelvic ring stability in patients operated for unstable pelvic ring fractures [23]. After 1 year five patients still had some pain in the symphysis. Due to artefacts, CT scans could not verify fusion in all cases. However, there were no indirect signs of non-union so it remains unclear why these patients reported persistent pain in the pubic area. Few studies have reported clinical results after symphysis plating in PGP patients [12,24], and it could be questioned whether fusion of the pubic symphysis is necessary in patients with PGP.

Non-specific PGP is thought to be a multi-factorial disorder with genetic, social, psychological, neuro-physiological and patho-anatomical factors involved in the pain syndrome [6]. SIJ fusion is used to treat these patients based on a biomechanical understanding of the disorder. Although it is difficult to evaluate whether the pain originates from the synovial joint or the surrounding ligaments, fusion will most likely, aside from stabilising the joint, also reduce the stress on the surrounding ligaments. Hence the positive results might be a consequence of greater SIJ stability. On the other hand, the placebo effect of surgery might also have had an impact on patient outcomes [25].

Because conservative treatment has proven effective for patients with PGP [26], this should be the first choice for therapy [6]. However some patients remain severely disabled with persistent pain despite appropriate conservative treatment [27]. Because of the possibility of complications, and a lack of randomized controlled trials, SIJ fusion needs to be further studied. Recently, new percutaneous devices for SIJ fusion have been introduced, and first reports [28-30] show a low complication rate together with a high fusion rate. These new techniques may reduce complications, however evidence of their efficacy has yet to be demonstrated.

## Conclusion

One year after open unilateral anterior SIJ fusion combined with symphysis pubis fusion, positive and significant changes in both physical function and pain were observed. Despite these positive results, this procedure was associated with adverse events and complications.

## Abbreviations

SIJ: Sacroiliac joint; PGP: Pelvic girdle pain; ODI: Oswestry disability index; VAS: Visual analogue scale; SSRD: Single subject research design.

## Competing interests

This study was supported by grants from the Norwegian Foundation for Health and Rehabilitation and Sophies Minde Ortopedi AS. The authors declare that they have no competing interest.

## Authors’ contributions

All authors have been involved in the planning of the study, data collection, data analysis and the writing. All authors read and approved the final manuscript.

## Pre-publication history

The pre-publication history for this paper can be accessed here:

http://www.biomedcentral.com/1471-2474/15/85/prepub
